# Helping Infants to Help: Cultural Variation in Maternal Scaffolding and the Development of Prosocial Behavior

**DOI:** 10.1037/dev0002159

**Published:** 2026-05-07

**Authors:** Carlo Vreden, Moritz Köster, Lucy Abbit, Prima Ainembabazi, Aileen Cook, Brenda Kakai, Precious Katusiime, Julia Landstedt, Sarah Mazari, Eunice Ndyareeba, Elizabeth Renner, Georgia Tuohy, Zanna Clay

**Affiliations:** 1 Department of Psychology, Durham University; 2 Child Development in Educational Contexts, DIPF | Leibniz Institute for Research and Information in Education, Frankfurt, Germany; 3 Institute of Psychology, University of Regensburg; 4 Department of Foundations of Education, Kabale University; 5 Department of Psychology, Northumbria University

**Keywords:** cross-cultural psychology, prosocial behavior, helping, socialization, scaffolding

## Abstract

Infants engage in a range of prosocial behaviors from a young age. Caregiver scaffolding, through encouragement and requests, plays a key role in shaping these early tendencies. However, how scaffolding practices vary with socialization goals across cultural contexts, and whether their effects on infant behavior are culturally specific, remains unanswered. In this study, we took a longitudinal, cross-cultural approach to examine variation in maternal socialization goals, scaffolding strategies, and infant helping at 16 and 22 months across three distinct sociocultural settings. A total of 273 infants participated in rural Uganda (*n* = 49; 24 females, 25 males), urban Uganda (*n* = 162; 76 females, 86 males), and in the United Kingdom (*n* = 62, 29 females, 33 males). We assessed maternal socialization goals via questionnaires, scaffolding strategies during a requested helping task, and infant prosocial behavior through both requested and spontaneous helping tasks. Scaffolding was more assertive in the Ugandan samples compared with those in the United Kingdom, where scaffolding was more deliberate. Maternal assertiveness positively predicted both spontaneous and requested helping at the individual level. However, at the group level, spontaneous helping rates were higher in Uganda than in the United Kingdom, whereas requested helping was higher in the United Kingdom than Uganda. These findings reveal important cross-cultural variation in how caregivers shape infant helping and point to a nuanced interplay between culturally specific caregiving practices and emerging prosocial behavior. Our study underscores the importance of examining both universal and context-specific pathways in the development of prosociality.

## Public Significance Statement

Mothers in different cultural contexts shape young children’s helping behavior in distinct ways, and these caregiving strategies influence how and when children help others. The findings highlight that although helping may be a human universal, children’s prosocial development is also influenced by individual parenting practices and broader cultural values and goals.

Humans are a remarkably prosocial species, helping others achieve goals from as early as their first birthday (Dahl, 2015). Following the seminal work of Warneken and Tomasello, who examined prosocial helping in 14-month-olds (2006, 2007), almost 2 decades of research has documented infants’ tendency to help others, such as handing back out-of-reach objects to an experimenter (e.g., Callaghan et al., 2011; Dunfield et al., 2011; Giner Torréns & Kärtner, 2017; Köster et al., 2019; Warneken & Tomasello, 2013; for a review, see Köster & Kärtner, 2019). Although infant helping appears to be a human universal, it is nonetheless influenced by social interactions with caregivers, who scaffold helping through their requests, encouragement, and praise as part of daily routines (e.g., Dahl et al., 2017; Hammond & Carpendale, 2015). This scaffolding promotes not only helping in response to adult requests but also spontaneous (i.e., unrequested) helping too (Köster et al., 2016).

Given the vast diversity of sociocultural contexts within which infants develop, understanding cross-cultural variation in caregiver scaffolding practices is thus important for understanding which factors shape infant helping. In a cross-cultural study across rural and urban Brazil and Germany, Köster et al. (2016) found striking variation in how mothers instructed their 18- to 30-month-old children to help them in a clean-up task. Brazilian mothers from a rural low-resource setting were highly assertive and insistent in how they engaged with their infants, placing high importance on obedience and fulfilling interpersonal obligations. Urban German mothers by comparison were more deliberate, giving explanations and pleas while framing the task as fulfilling personal needs. Interestingly, Brazilian mothers from an urban, high-resource context displayed both styles, being more assertive than German mothers but more deliberate than rural Brazilian mothers. Across sites, assertive scaffolding predicted higher infant compliance with maternal requests to help. However, when tested on a spontaneous helping task (handing back an accidentally dropped or out-of-reach objects to an experimenter), assertive scaffolding only positively predicted helping in rural Brazil, while deliberate scaffolding was predictive in urban Germany. These findings suggest that although assertive scaffolding is effective in fostering infant obedience, there are nonetheless culture-specific ways in which maternal scaffolding influences infant helping. It should be acknowledged that although deliberate scaffolding in particular often involves encouragement or praise, it does not necessarily entail external rewards. This distinction is important because extrinsic, material rewards have been shown to reduce rates of instrumental helping in 20-month-old infants compared with those who received no response or a social reward, that is, praise, to their helping (Warneken & Tomasello, 2014). Thus, although explicit scaffolding may facilitate infant prosociality, prosocial motivation appears to be a robust trait in early development, something that has also been documented in eye-tracking studies (e.g., Hepach et al., 2012). In this respect, maternal scaffolding appears to largely support, but also occasionally hinder, infant prosociality and likely does so in culture-specific ways.

Cross-cultural variation in maternal scaffolding is aligned with maternal socialization goals, which are culturally variable goals for child development based on environmental demands and cultural norms (Keller & Kärtner, 2013). Two broad prototypes of socialization goals have emerged as relevant for prosocial behavior development: an autonomous prototype, typically found in Western, urban, high-resource populations, which emphasizes independence and self-sufficiency, and a relational prototype prevalent in non-Western, rural, low-resource, which emphasizes interpersonal dependence, harmony, and obedience (Kagitcibasi, 2005; Keller, 2007). These socialization goals influence when behaviors, such as helping, are expected to emerge and how parents scaffold their development. The assertive scaffolding that Köster et al. (2016) observed in rural Brazilian mothers aligns with relational socialization goals, which focus on obedience and fulfilling interpersonal responsibilities. By contrast, the deliberate scaffolding observed in urban German mothers is characteristic of autonomous socialization goals, emphasizing the personal choice to help. Similar cultural variation in maternal socialization goals was found in a comparison of urban German (Berlin) and urban India (Delhi), with Berlin mothers placing greater emphasis on autonomous socialization goals and Delhi mothers emphasizing stronger relational socialization goals of prosociality and obedience (Kärtner et al., 2010). Across sites, relational socialization goals, specifically obedience, correlated with infant prosocial behavior, while autonomous socialization goals had no effect in either site. It should be noted that the prosocial behavior here was comforting of an adult feigning distress, that is, empathic prosocial behavior, the development of which is at least somewhat distinct from instrumental helping (Dunfield et al., 2011). However, combined with Köster et al.’s study, these findings emphasize that cultural contexts shape both socialization goals and practices for prosocial behavior and that some of the effects of these on infant helping are stable across cultures, while others follow culture-specific pathways.

Differences in socialization goals and practices for prosocial behavior—as personal choice or interpersonal obligation—reflect broader cultural conceptualizations of prosociality, which extend beyond infancy into broader cultural patterns (Miller et al., 1990). Beyond cultural values, these differences should also be considered in the context of environmental demands, which in turn can shape cultural values. The different degrees to which caregivers depend on their children’s help likely also contribute to how helping is socialized and develops. For instance, in low-resource contexts wherein households may depend on subsistence farming, lack running water or electricity, or have less caregiver support, caregivers may depend on the early involvement of young children to complete simple household tasks, such as gathering firewood, preparing food, or fetching water. In such settings, children’s obedience in completing communal tasks may be more heavily emphasized with their involvement being expected from toddlerhood (Lancy, 2020; Nsamenang, 1992). By comparison, in high-resource contexts, which characterize the European and North American demographic of most infant helping studies thus far, caregivers tend to place more emphasis on children’s personal freedom, with helping viewed more as personal choice and subject to more praise. In a recent study comparing infants from a rural, subsistence farming community in Uganda and infants from a suburban context in the United Kingdom, Buryn-Weitzel et al. (2025) found that Ugandan infants were indeed more likely and quicker to help an adult in need than were U.K. infants. Moreover, over 85% of mothers in Uganda expected their 18-month-old child to help them or others in everyday life compared with only 35% of English mothers. This result illustrates the different cultural demands on infants to behave prosocially, which likely shape how insistently mothers scaffold these behaviors. Although this study was based on a rural Ugandan sample (as compared with an urban U.K. sample), urban non-Western contexts are also informative for exploring how shifting environmental demands can influence cultural values and the development of helping behavior. With the move away from subsistence farming, urbanization can rapidly change the ecological demands a family faces, including the need and expectations for children’s helping, such that help from children with farming and household tasks becomes less essential. As such, differences in infant helping and maternal expectations within urban settings could reflect both cultural shifts and ecological factors, themselves interrelated.

In the present study, we expand upon previous work and address these issues, including the relative paucity of cross-cultural work in this area by investigating maternal scaffolding, socialization goals, and their link to infant prosocial behavior across three different sociocultural settings at two time points. To assess if maternal scaffolding is linked with infant-requested helping, 16-month-old infants and their mothers completed a scaffolding task in which their mother asked them to help place objects in a box (Köster et al., 2016). Infants also completed a classic out-of-reach helping task with an experimenter (Warneken & Tomasello, 2006) at 16 and 22 months to test if maternal scaffolding also influenced spontaneous helping (Dahl, 2015) and whether this effect would be developmentally consistent.

We tested infants from three sites: rural Uganda, urban Uganda, and urban/suburban United Kingdom. Previous work has shown that this rural Ugandan site represents a prototypical low-resource, subsistence-based farming context wherein mothers prioritize relational socialization goals and conceptualize prosociality as a matter of interpersonal obligation (Holden et al., 2022). By comparison, the U.K. site represents a high-resource, Western urban setting wherein prosociality is more linked to personal choice, aligning with autonomous socialization goals. Another recent study using comparable samples in rural Uganda and the United Kingdom showed site differences in maternal socialization of prosociality and prosocial outcomes at 18 months (Buryn-Weitzel et al., 2025): In this study, mothers from the United Kingdom reported using a greater variety of scaffolding strategies for infant helping (such as asking, demonstrating, or rewarding) than rural Ugandan mothers did, and this variety was associated with higher spontaneous helping in a classic out-of-reach task. In rural Uganda, mothers reported less requesting, demonstrating, and rewarding of helping, and these strategies were not linked with infant spontaneous helping. Nevertheless, Ugandan mothers were more likely to expect their child to help, and their infants were more likely to help overall. These somewhat mixed findings suggest that it may be the quality of how mothers ask and instruct helping rather than how much they do so. It is relevant to note that Buryn-Weitzel et al. (2025) investigated maternal scaffolding via self-report only; therefore, it is unclear to what extent scaffolding practices differed cross-culturally.

In this study, we addressed this question by examining cross-cultural variation in maternal scaffolding behavior and its relation to infant prosocial development. To investigate the potentially competing cultural influences of autonomy and relatedness (Kagitcibasi, 2005; Köster et al., 2016) and the broader influences of urbanization on parenting practices, we included data from three cultural settings: rural Uganda, urban Uganda, and urban United Kingdom. Despite many ecological and cultural similarities in urban and rural Uganda, urbanization also signifies many key lifestyle changes, including increased access to formal education and (Western) media, engagement in wage economy, and globalization, enhancing the scope for intracultural differences. Previous work on toddler helping in rural and urban Brazil (Köster et al., 2016) suggests that urbanization may impact the nature of parental scaffolding behaviors and infant helping, something we set out to test.

We tested the main hypothesis that infant helping is shaped by the social learning environment, which varies across cultural caregiving contexts. In line with previous research, we predicted that mothers from (rural and urban) Uganda would exhibit more relational socialization goals than U.K. mothers (Holden et al., 2022) and have higher and earlier expectations for infant helping (Buryn-Weitzel et al., 2025). By comparison, U.K. mothers would be more autonomous in their socialization goals and have less expectations for infant helping. If urbanization results in a potentially closer alignment toward urbanized Western values and lifestyles, mothers from our urban Ugandan sample may be therefore expected to place greater emphasis on autonomy than the rural Uganda setting.

Regarding maternal scaffolding, we predicted maternal strategies to vary across sites with higher assertive scaffolding (serious and insistent requesting) in Uganda as compared with higher deliberate scaffolding (reasoning and pleading) in the United Kingdom. If our within-country results follow similar patterns as have been previously reported in urban and rural Brazil (Köster et al., 2016), greater deliberate scaffolding might be expected to occur in the urban versus rural setting as well.

For infant compliance to help (i.e., following maternal requests), we predicted that assertive scaffolding would positively predict requested helping across sites, as infants should comply more when requests emphasize obligation rather than personal choice (Köster et al., 2016). Similarly, assertive scaffolding was expected to positively predict spontaneous helping (toward an experimenter) across sites, as mothers who emphasize prosociality as an obligation should foster spontaneous infant prosocial behaviors even toward strangers, without explicit prompting (Dahl et al., 2022). However, the strength of this link could vary across cultural contexts according to variation in infant socialization: Higher assertive scaffolding (as predicted in Uganda) could lead to higher helping there. However, other studies have found conversely higher rates of spontaneous helping in urban Western infants (e.g., Callaghan et al., 2011; Köster et al., 2016; although see Giner Torréns & Kärtner, 2017), despite lower levels of assertive scaffolding; thus, spontaneous helping rates might be higher in the United Kingdom.

Last, if deliberate scaffolding represents a culture-specific strategy, we predicted that it would only be linked with higher rates of requested and spontaneous helping in the United Kingdom, but not in Uganda, where deliberate scaffolding would be rare or absent.

## Method

### Participants and Research Sites

A total of *N* = 273 mother–infant dyads participated across three research sites (rural Uganda, urban Uganda, the United Kingdom) at the first time point at 16 months, with *N* = 221 returning for the follow-up time point at 22 months. Participants were part of a larger, longitudinal cohort followed throughout the first 2 years of life and had completed at least one testing session prior to this study. In the United Kingdom, participants were recruited through online adverts and playgroups, in rural and urban Uganda at village meetings, with the help of local community research assistants, village health teams, and through word of mouth. Culturally appropriate reimbursements were given to all participants (as household goods in rural Uganda, cash rewards in urban Uganda, or shopping vouchers in the United Kingdom), based on advice from local teams and partners. See Table 1 for infant demographic information.[Table tbl1]

#### Rural Uganda

The rural Ugandan site is located in Nyabyeya Parish, Masindi district, in Western Uganda on the edge of Budongo forest. Background questionnaires revealed that this was a majority subsistence farming community with some cash crops and low levels of wage labor. Most mothers had only primary school education, as secondary schools are far away or unaffordable. Households typically consist of multiple families meaning that childcare is often distributed between relatives, including other, older children as caregivers for infants. Due to the high work demands of a subsistence farming lifestyle, children are expected to help with household chores from an early age.

#### Urban Uganda

The urban Ugandan site, Mbarara, is one of the country’s largest cities and located in Southwestern Uganda. Background questionnaires revealed that engagement in wage labor is common and access to education is better, so the majority of mothers had at least a secondary school education and a profession outside of the home. Children are still socialized to engage in household chores from an early age but are not seen as a labor force to the same extent as in rural Uganda, meaning that early helping is expected but practically less essential for the running of the household.

#### United Kingdom

The U.K. sample represents a Western-rearing environment classically studied in developmental research: Participants came from a small city, Durham, and surrounding towns in the northern United Kingdom. Background questionnaires revealed that all participating mothers had a postsecondary education and at least one parent within the household held formal employment. Although children are encouraged to help from early on, they are not relied on for the upkeep of the household. Due to the overall pervasive impact of urbanization and mobility in the United Kingdom, we did not include a rural versus urban comparison in the United Kingdom. Cultural and ecological differences between these settings can be expected to be much smaller than in Uganda and their nuances more difficult to effectively interpret, particularly given the broader goals of the study.

### Ethics

Ethical approvals were obtained from the Department of Psychology at Durham University, the Ugandan Virus Research Institute (GC/127/20/08/788), and the Ugandan National Council for Science and Technology (SS596ES). All mothers gave their informed consent for their participation and that of their child. In the United Kingdom, written consent was obtained through an online questionnaire, during which participants also filled in demographic information. In Uganda, due to limited literacy of some mothers, information sheets were read out in local languages by local researchers, and mothers gave verbal consent, which was video recorded, and also signed with either their signature or a thumbprint (an accepted form of signing official documents).

### Procedure and Materials

Testing took place at the participant’s home in rural Uganda and in the United Kingdom and in a primary school in urban Uganda (home visits were not feasible due to the larger sample size). In rural Uganda, testing was completed outdoors in the family compounds, where families spend most of the day, due to limited space/light inside. In urban Uganda and the United Kingdom, testing was completed indoors. Trials were recorded using two Panasonic HC-VX870 4 K cameras: one mounted on a tripod to capture the whole scene and one handheld to follow the infant’s movement. See Figure 1 for setup photos.[Fig fig1]

#### Questionnaires

To measure mothers’ socialization goals, we used the adapted version of Keller’s (2007) Socialization Goals Questionnaire as has been previously used in this rural Uganda setting (Holden et al., 2022). This questionnaire consists of eight items relating to important developmental goals for children in the first 3 years of life (e.g., “During the first 3 years of life, it is really important that children learn to obey elders”), to which mothers were asked to state their (dis-)agreement on a 5-point Likert scale from strongly disagree to strongly agree. There were five items relating to relational socialization goals and three items relating to autonomous socialization goals. Additionally, we asked mothers (as part of a questionnaire adapted from one developed by a collaborator, K. Slocombe and team, on socialization practices) if they expected their child to help them or others (“I expect my child to help me or others,” to which they could agree or disagree). Translation of questionnaire items was completed using independent forward and backward translations by local researchers, based on the studies by Holden et al. (2022) and Buryn-Weitzel et al. (2025).

In Uganda, following procedures by Holden et al. (2022), questionnaires were read aloud by local research assistants or played from a recording in the mother’s preferred language to which the mother gave her verbal responses as noted down by the research assistant. In rural Uganda, questionnaires were administered in Alur, Lugbara, Ugandan Swahili, and Runyoro, in urban Uganda in Runyankole. In the United Kingdom, questionnaires were completed online via Qualtrics before the visit.

#### Requested Helping (16 Months)

To assess maternal scaffolding and infants’ responses to it, we used a requested helping task adapted from Köster et al.’s (2016) study. Mother and infant were seated in one corner of a playmat. In the other three corners, the experimenter placed a cup, a toy shovel, and a small sieve along with an empty box placed in the center of the playmat. Mothers were instructed to ask their child to put the three items in the box however they normally would and to not actively help their child or to touch the box or objects. The mother was given 3 min to request the child’s help. If unsuccessful within the given time, the mother was asked if she would like to try for another minute. The trial ended either when all objects were in the box or after 3 (plus 1) minutes. If mothers did not follow instructions, the experiment was repeated at a later time with other tasks placed in between. Requested helping, unlike spontaneous helping (see below), was only assessed at 16 months as maternal scaffolding and infant-requested helping are expected to be relatively stable across this short time span and by 22 months, at ceiling. Spontaneous helping, which undergoes more rapid developmental change across this age window, was assessed at 16 and 22 months to examine how maternal strategies affect its development concurrently and prospectively.

#### Spontaneous Helping (16 and 22 Months)

Spontaneous helping was assessed at 16 and 22 months using two classic out-of-reach scenarios (Warneken & Tomasello, 2006), a “cups” task and a “bottle” task, using culturally appropriate items common to all three sites.

In the cups task, the experimenter sat behind a small table with six plastic cups placed in two rows of three. The experimenter began by looking at the cups and saying, “Look, I am going to build a tower with my cups,” and started to place one cup on top of the other. After stacking two cups, they pretended to accidentally drop the third cup and made a surprised noise (“Oops!” in the United Kingdom, “Eh!” in Uganda). They then looked at the item on the floor for 15 s, reaching for it with their outstretched hand, while making effortful sounds. For the next 15 s, they alternated their gaze between the dropped object and the infant while still reaching for it. If the infant handed them the object, the experimenter continued building their cup tower without thanking the infant, so as not to encourage them. If the infant did not return the object after 30 s, the experimenter stood up and picked it up themselves. After the third cup, the experimenter stacked the fourth cup but then dropped the fifth and sixth cups using the same protocol as above. This meant that each infant had three opportunities to help pick up a dropped cup for the experimenter.

In the bottle task, the procedure was the same as in the cups task, except that the scenario involved picking up and dropping a plastic drink bottle and its lid. The task began with the experimenter saying, “I’m very thirsty, I am going to have a drink,” after which they unscrewed the lid from a plastic bottle and pretended to take a sip. They then replaced the lid and dropped the bottle. In the second trial, they said, “I’m still thirsty, I am going to have another drink.” However, in this case, they only dropped the bottle lid, keeping the bottle in their hand.

The order of tasks was kept the same for all infants, who participated in all tasks, without counterbalancing. This was necessary to accommodate other tasks completed in the same testing session not presented in this study.

### Coding and Data Preparation

#### Questionnaires

The Socialization Goals Questionnaire was scored by calculating the mean Likert scale rating for the three autonomous and five relational items separately. Because previous research has shown that Ugandan mothers often only used the extremes of the 1–5 scale (*strongly agree/disagree*), while U.K. mothers were more likely to use the full scale. We calculated a socialization goals difference score based on subtracting the mean relational score from the mean autonomous score, as has been done before with rural Ugandan and U.K. participants (Holden et al., 2022). Potential values ranged from −4 to 4, with negative values indicating more relational and positive values more autonomous socialization goals. The “expecting infants to help” question was scored as a binary yes/no response.

#### Maternal Scaffolding and Requested Helping

Within the requested helping task, we coded maternal scaffolding using a coding scheme adapted from Köster et al.’s (2016) study, focusing on assertive (serious and insistent requesting) and deliberate (asking, pleading, and giving explanations) maternal scaffolding behavior. Each mother received one separate score for each behavior, based on the coder’s overall scoring of the trial. Assertive and deliberate scaffolding were each rated on a 4-point scale from 0 (*not at all important*) to 3 (*very important*). Interobserver reliability between two coders was carried out for 15% of the data (see below for results).

Assertive scaffolding measured how important the mother considered the completion of the task at hand. Coding was based on the seriousness and insistence of the instructions, with a focus on the mother’s tone, repetition of requests, and responses to off-task behavior of the infant. Interobserver reliability was high (Cohen’s κ = .81).

Deliberate scaffolding measured how important the mother’s instructions considered the infant’s autonomy and understanding of the task to be. It was measured based on the relative frequency of the mother’s use of asking, pleading, and giving justifications during the trial (e.g., “Can you get the cup for me?” “Please bring the cup so we can drink!”). Questions such as “Where is the cup?” were not included as examples of deliberate scaffolding, as they may have primarily been used to grab the infant’s attention. Interobserver reliability was very high (Cohen’s κ = .96).

Infant-requested helping was scored for how many items they successfully put in the box. If infants placed an item in the box and later during the trial took it out again, this item was still counted as a success. Infants could receive a maximum score of three items.

#### Spontaneous Helping

For each task, we scored each item which the infant picked up and returned to the experimenter who was reaching for it (three items for the cups task and two items for the bottles task). This included picking up the object and putting it in the experimenter’s outstretched hand, holding it out for them to take, and putting it on the table. We did not consider placing the dropped cup on the cup tower as helping, nor putting the lid on the bottle, because it is not possible to distinguish between these actions as helping or simply completing an action. In total, infants could receive a score of 5 (3 for the cups task, 2 for the bottle). Interobserver reliability was very high (Cohen’s κ = .93).

### Statistical Analysis

#### General Approach

All analyses were conducted in R (Version 4.4.1). For generalized linear mixed models (GLMMs; Baayen, 2008), we used the lme4 package (Bates et al., 2015). All models were first compared with a null model without the predictor fixed effects but with the same random effects and error structure as the full model using likelihood ratio tests (Dobson, 2002). Following a significant full-null model comparison, we examined the contribution of fixed effects using the drop1 function. If this showed that an interaction term from the full model was not significant, we continued with a reduced main-effects model into which the interaction terms were instead entered as independent effects but otherwise retaining the structure of the full model. This reduced main-effects model was then examined again using the drop1 function. Post hoc contrasts between individual predictor levels were conducted using the emmeans package (Lenth, 2022). This study was not preregistered. Full model estimates can be found in the Supplemental Materials. Data and analysis code for this study are available at Vreden and Clay’s (2026) study.

#### Questionnaires

To test for site differences in maternal socialization goals, we used a Kruskal–Wallis test on the −4 to 4 socialization goals difference score by site (rural Uganda, urban Uganda, the United Kingdom), followed up with post hoc Dunn tests with Holm *p* adjustment for multiple comparisons to establish significance between individual sites. To test for site differences in maternal expectations for infant helping, we used a binomial GLMM with maternal expectation to help (yes, no) as the outcome variable. We entered an interaction of site (rural Uganda, urban Uganda, the United Kingdom) and infant age (16 months, 22 months) and a random intercept for participant ID.

#### Maternal Scaffolding of Requested Helping

To test for site differences in maternal scaffolding styles within the requested helping task, we performed Kruskal–Wallis tests on the assertive and the deliberate maternal scaffolding score (both 0 and 3) by site (rural Uganda, urban Uganda, the United Kingdom). Significant Kruskal–Wallis tests were followed up with post hoc Dunn tests with Holm *p* adjustment for multiple comparisons to establish significance between individual sites.

#### Effect of Scaffolding on Requested and Spontaneous Helping

Table 2 shows the model structures of the binomial GLMMs used to test for the effects of assertive and deliberate scaffolding on requested and spontaneous helping. Scaffolding scores were 0 to 3. An analysis of discrete proportions in a binomial GLMM is possible using a two-column matrix with the number of successes and failures per individual as the response (Baayen, 2008). Therefore, requested and spontaneous helping were entered into the models as successes–failures matrices (e.g., placed two items in the box, did not place one item in the box for requested helping; helped on two trials, did not help on three trials for spontaneous helping). Deliberate scaffolding was displayed by all U.K. mothers (all received a rating of at least 1 but very rare among Ugandan mothers [only 17% showed any deliberate scaffolding and none received a score higher than 2]). As this resulted in statistical separation of the data between sites, we only analyzed assertive scaffolding across all three samples. For deliberate scaffolding, as this has been shown to be linked with infant helping in Western contexts (Köster et al., 2016), we analyzed its effect in the United Kingdom. We were unable to do so in Uganda due to its very limited occurrence, also corresponding with prior research, which found no positive link between deliberate scaffolding and helping in non-Western contexts (Köster et al., 2016). Due to separation and restricted distribution of the deliberate scaffolding data (as described above), we did not directly compare the two forms of scaffolding in one model. The requested helping models were based on the 16-month data only, as this was not completed at 22 months.[Table tbl2]

## Results

### Maternal Socialization Goals and Maternal Expectations for Infant Helping

Maternal socialization goals differed between sites, H(2) = 95.94, *p* < .001. As predicted, U.K. mothers’ socialization goals were the most autonomous compared with those of the rural Ugandan mothers (*Z* = 3.83, *p* < .001) and urban Ugandan mothers (*Z* = 9.60, *p* < .001). Although Ugandan mothers were more relational than U.K. mothers, mothers from the urban Ugandan site emphasized relational socialization goals more than those from the rural site (*Z* = 4.30, *p* < .001).

Regarding maternal expectations for infant helping, the full-null model comparison was significant, χ^2^(5) = 26.70, *p* < .001, but the interaction between site and age was not, χ^2^(2) = 5.16, *p* = .076. Continuing with the main-effects model, there was a main effect of site on maternal expectations for infant helping, χ^2^(2) = 19.26, *p* < .001. As predicted, mothers from rural Uganda (estimate ± *SE* = 5.30 ± 1.19, *z* ratio = 4.45, *p* < .001) and urban Uganda (estimate ± *SE* = 6.68 ± 1.16, *z* ratio = 5.78, *p* < .001) were more likely than U.K. mothers to expect their child to help them or others. No such difference was found between the two Ugandan sites (estimate ± *SE* = 1.38 ± 1.05, *z* ratio = 1.31, *p* = .391). There was no significant effect of infant age on maternal expectations of helping, χ^2^(1) = 1.10, *p* = .294.

Table 3 shows maternal socialization goals at each site, calculated as a difference score based on Holden et al.’s (2022) study, as well as the percentage of mothers who expected their child to help them or others at 16 and 22 months.[Table tbl3]

### Maternal Scaffolding of Infant-Requested Helping

Maternal scaffolding styles differed between sites, assertive scaffolding: H(2) = 34.73, *p* < .001; deliberate scaffolding: H(2) = 182.97, *p* < .001. As predicted, Ugandan mothers (rural and urban) were more assertive than U.K. mothers: Rural Ugandan mothers were more assertive than mothers from urban Uganda (*Z* = 2.24, *p* < .001) and the United Kingdom (*Z* = 5.59, *p* < .001), and urban Ugandan mothers were more assertive than U.K. mothers (*Z* = 4.71, *p* < .001). U.K. mothers used the highest levels of deliberate scaffolding compared with mothers from rural Uganda (*Z* = 9.10, *p* < .001) and from urban Uganda (*Z* = 13.40, *p* < .001). Although deliberate scaffolding was overall very rare in Uganda, rural Ugandan mothers used more deliberate scaffolding than urban Ugandan mothers (*Z* = 1.60, *p* < .001). See Figure 2.[Fig fig2]

### Assertive Scaffolding and Requested Helping

The full-null model comparison was significant, χ^2^(5) = 54.56, *p* < .001, but the interaction between assertive scaffolding and site was not, χ^2^(2) = 3.23, *p* = .199. Continuing with the main-effects model, we found a main effect, as predicted, of assertive scaffolding on requested helping, with higher maternal assertive scaffolding predicting greater infant-requested helping across sites, estimate ± *SE* = 0.82 ± 0.26, χ^2^(1) = 10.31, *p* = .001. Analysis of infant-requested helping revealed site differences, χ^2^(2) = 50.42, *p* < .001: U.K. infants showed more requested helping than infants from rural Uganda (estimate ± *SE* = 3.77 ± 0.72, *z* ratio = 5.23, *p* < .001) and urban Uganda (estimate ± *SE* = 3.55 ± 0.58, *z* ratio = 6.07, *p* < .001). No such difference was found between the two Ugandan sites (estimate ± *SE* = 0.23 ± 0.50, *z* ratio = 0.45, *p* = .893). See Figures 2 and 3.[Fig fig3]

### Deliberate Scaffolding and Requested Helping

The full-null model comparison was not significant, χ^2^(1) = 0.02, *p* = .894, indicating no effect of deliberate scaffolding on infant-requested helping in the United Kingdom.

### Assertive Scaffolding and Spontaneous Helping

The full-null model comparison was significant, χ^2^(7) = 173.68, *p* < .001, but neither the interaction between assertive scaffolding and age, χ^2^(1) = 0.16, *p* = .685, nor the interaction between assertive scaffolding and site, χ^2^(2) = 1.44, *p* = .487, were significant. Continuing with the main-effects model, there were main effects of assertive scaffolding, site, and age. As predicted, assertive scaffolding predicted more infant spontaneous helping across sites, estimate ± *SE* = 0.39 ± 0.13, χ^2^(1) = 8.52, *p* = .003.

Additionally, spontaneous helping differed across sites, χ^2^(2) = 17.30, *p* < .001, where rural Ugandan infants showed more spontaneous helping than urban Ugandan (estimate ± *SE* = 0.98 ± 0.27, *z* ratio = 3.69, *p* < .001) and U.K. (estimate ± *SE* = 1.32 ± 0.34, *z* ratio = 3.97, *p* < .001) infants. There was no difference in spontaneous helping between infants from urban Uganda and the United Kingdom (estimate ± *SE* = 0.34 ± 0.26, *z* ratio = 1.30, *p* = .397). Last, with respect to age, infant helping increased with age (from 16 to 22 months) across sites, estimate ± *SE* = 1.17 ± 0.10, χ^2^(1) = 131.62, *p* < .001. See Figures 4, 5, and 6.[Fig fig4][Fig fig5][Fig fig6]

### Deliberate Scaffolding and Spontaneous Helping

The full-null model comparison was significant, χ^2^(3) = 17.76, *p* < .001, but the interaction between deliberate scaffolding and age was not, χ^2^(1) = 1.58, *p* = .209. Continuing with the main-effects model, deliberate scaffolding did not significantly increase spontaneous helping, χ^2^(1) = 0.10, *p* = .758.

## Discussion

Despite large differences in the cultural and ecological environments that infants experience across the globe, converging research indicates that the emergence of prosociality in early human development may be culturally ubiquitous (e.g., Callaghan et al., 2011). Our study supports the hypothesis that infant helping is nevertheless influenced by early social experiences with caregivers, which are shaped by broader sociocultural norms and values (Köster et al., 2016). Comparing across three cultural settings, rural and urban Uganda as well as the United Kingdom, we found that the way caregivers instruct their children to help, which is known as scaffolding, corresponded with mothers’ broader cultural attitudes and values related to collectivism, obedience, and autonomy as well as their expectations about children’s prosociality. Assertive scaffolding, which involves serious and insistent caregiver requests, was highest in our rural Ugandan setting, where infant helping is not only culturally valued but also, practically speaking, important. Deliberate scaffolding, which involves pleading and encouraging, was most common in the United Kingdom; however, only assertive maternal scaffolding predicted higher infant spontaneous and requested helping across all sites, suggesting that it is an effective strategy for fostering prosocial behavior. By relating maternal socialization goals, cultural values, and infant helping behavior across distinct caregiver settings, our study highlights the important role that early sociocultural experiences play in shaping the development of human prosociality, one of our most important species attributes.

A previous study (Köster et al., 2016) proposed that mothers from relational cultural contexts, which emphasize interpersonal dependence, harmony, and obedience (Kagitcibasi, 2005; Keller, 2007), tend to be more assertive in their scaffolding style, such as insisting on their infant’s help as a form of obedience. By comparison, mothers from autonomous cultural contexts, which emphasize independence and individuality, such as the industrialized West, show more deliberate scaffolding styles, such as pleading and explaining. In autonomous contexts, helping is seen as a personal choice motivated by an understanding of others’ states and needs, rather than a societal obligation (Miller et al., 1990).

Supporting these predictions and in line with related research (Buryn-Weitzel et al., 2025; Holden et al., 2022), Ugandan mothers from both sites strongly emphasized relational socialization goals, which were associated with a more assertive scaffolding style and a higher expectation of infant helping than in the United Kingdom. By comparison, U.K. mothers displayed autonomous socialization goals, along with more deliberate scaffolding strategies and less expectation for infant helping. In previous research, mothers from rural Brazil, a relational context, were similarly more assertive than urban, autonomous-relational Brazilian mothers, with both urban and rural Brazilian mothers more assertive than a German sample (Köster et al., 2016), who were more deliberate in how they instructed their infants to help. Taken together, our results support the view that cultural contexts shape caregiver’s socialization goals, which in turn shape how they scaffold their children’s emerging prosocial behavior.

Parental socialization goals are also closely related to ecological and demographic factors. In rural low-resource settings, like the rural Ugandan site in our study, large households, limited modern conveniences, and subsistence farming demand cooperation in daily chores, rendering children’s early and effective help both necessary and expected (Crittenden, 2016). This contrasts with urban and/or Western environments (where strong contrasts between rural and urban lifestyles are less visible), such as our U.K. suburban setting, where engagement in wage economy, greater dependence on externally sourced food, and access to conveniences may render infant helping less essential (Hammond & Brownell, 2018). Although we did not formally analyze the impacts of detailed ecological variables relating to urban and rural living, background information collected as part of the overall longitudinal project (e.g., access to electricity and television, household structure, living arrangements) aligns with these sites’ characterization as being representative or rural and urban lifestyles. Systematically identifying which features, or clusters of features, of urbanized environments shape prosocial development presents an avenue for future research. However, already, results from our study highlight the dual importance of cultural and ecological factors in shaping caregiver behavior and the broader influence that local living conditions have on caregiver strategies and developmental outcomes.

Although deliberate scaffolding was strongly associated with the U.K. caregiving context (98% of all U.K. mothers used deliberate scaffolding strategies), rates of deliberate scaffolding in our rural Ugandan sample were significantly higher than the urban setting (31% rural; 14% urban). This contrasts with predictions that socialization practices that foster personal autonomy should be more prevalent in urban contexts (Keller, 2007). We interpret this discrepancy as highlighting the nuances of how autonomy is perceived and valued across different cultural settings. For instance, a socialization strategy toward cooperative autonomy has been documented in some forager populations (Boyette & Lew-Levy, 2020; Endicott, 2011), where obligations to the group are emphasized along with the freedom from coercion against one’s personal choice. This translates to socialization practices that allow for some personal autonomy, such as on-demand breastfeeding, little interruption or guiding of infant exploration, or unsupervised movement of young children in peer-aged groups (Hewlett & Roulette, 2016; Hewlett et al., 1998). While these socialization practices are more common in forager populations compared with agriculturalist ones (Boyette & Hewlett, 2017; Hewlett et al., 1998), they may still be more common in rural settings compared with urban ones, as we found in Uganda. These intracultural differences emphasize the importance of not relying on West versus rest dichotomies and assuming all non-Western samples to perform in similar ways.

A key hypothesis underlying cross-cultural studies of prosocial development is the existence of culture-specific pathways for how maternal socialization strategies shape infant prosociality (Köster et al., 2016). Based on previous findings from a rural relational setting (in Brazil) and an urban Western setting (Germany), we predicted that assertive scaffolding would predict infant helping in rural Uganda, whereas deliberate scaffolding would predict higher infant helping in the United Kingdom. Although both assertive scaffolding and infant spontaneous helping were both highest in rural Uganda, our overall results did not strongly highlight culturally specific pathways given that assertive scaffolding positively predicted spontaneous helping across all sites and time points, while deliberate maternal scaffolding did not predict infant helping in the United Kingdom.

It is not clear why we were unable to replicate these culturally specific effects. Nevertheless, given that prosocial behavior motivations change with age (Dahl & Paulus, 2019) and that other related motor and social skills also change during this time, there are two potential methodological differences relating to infant age and coding that may have affected the patterns of results. In our study, we tested infants at 16 and 22 months, whereas Köster et al. (2016) tested 18- to 30-month-olds, with a mean age of 24 months. Furthermore, their coding was based on how quickly or hesitantly infants complied with maternal instructions, whereas our coding was based on the number of objects the infant successfully moved to their correct place. A further possible explanation may be that emphasizing the needs of others, through deliberate scaffolding, only fosters infant helping in combination with an emphasis on interpersonal responsibility and compliance, through assertive scaffolding. This could explain why helping was highest in rural Uganda, where mothers used both strategies. Further research is needed to disentangle the relationship between different maternal socialization strategies and how they influence infant behavior. This research should also acknowledge cultural diversity within and between settings which share broader characteristics. For instance, although both our study and that of Köster et al. compared non-Western rural and urban sites, these differ significantly in cultural and ecological factors, which might further explain the mixed findings relating to culturally specific links and cultural variation.

Regarding infant helping, we observed higher rates of spontaneous helping in rural Uganda compared with those in the United Kingdom. This corresponds with results from a recent study on which higher rates of helping in rural Uganda were found than in the suburban United Kingdom (Buryn-Weitzel et al., 2025) but is in contrast with previous studies that revealed no cultural differences in helping frequency (e.g., Callaghan et al., 2011; Köster et al., 2016). As noted, it could be that infants in rural Uganda are socialized toward prosociality based on interacting cultural and ecological pressures, where the constraints of communal living in a low-resource subsistence setting reinforce the importance of interpersonal obligation (Miller et al., 1990).

Like Köster et al. (2016), we also found that infants in a Western autonomous context (the United Kingdom) showed higher levels of requested helping than those in relational settings (Uganda). One interpretation for this is that U.K. infants may have been more familiar with this kind of task given that object-based activities, like our clean-up task, are a common form of mother–infant play in autonomous contexts. By comparison, Ugandan infants are socialized to comply with more serious helping requests in everyday life. This may explain why they helped more in the out-of-reach helping tasks that we used to test for spontaneous helping, which may more closely resemble real-world helping experiences from their daily routines (e.g., Alcalá et al., 2014; Giner Torréns & Kärtner, 2017; Ochs & Izquierdo, 2009). The difference in spontaneous helping between the rural and urban Ugandan sites also supports this: Spontaneous helping was higher in rural than urban Uganda, reflective of both ecological and socialization differences—mothers in rural Uganda reported higher expectations for infant helping and used more assertive scaffolding than those in urban Uganda (a higher caregiver emphasis on the obligation to help and an environment where helping is not only expected but needed to complete daily communal tasks may account for this variation). This underscores the importance of ecological validity when designing paradigms that are administered across groups, as different socialization environments may shape distinct helping behaviors that are more accurately reflected in specific task designs. Further observational work on infants’ naturalistic socialization experiences combined with ecologically/culturally grounded experimental work is needed to test this hypothesis.

While this study has contributed important new insights, there are some limitations that require further work to address. In particular, future research is needed to identify the directionality of the relationship between maternal sociality and infant helping, that is, the extent to which infants’ own developing prosociality might influence maternal scaffolding style. Experimental work that may shed further light on this could build on Dahl et al.’s (2022) methodology to manipulate the kind of scaffolding mothers use during tasks. Moreover, we did not examine the potential combined or interactive effects of assertive and deliberate scaffolding because of their markedly different distributions across sites. To better understand their unique and complementary effects, future research in samples wherein both styles are more evenly found could investigate how these two forms of scaffolding jointly contribute to prosocial development. In addition, although difficult to standardize effectively, another further direction would be to examine infants’ spontaneous helping toward their mothers, in addition to experimenters who are commonly the recipients in experimental studies. Last, although we found some commonalties between socialization influences and developmental outcomes across three cultural settings, we recommend caution in interpreting this as evidence of cultural universals. This is not a limitation per se but rather highlights the need for additional cross-cultural but also, and perhaps especially, intracultural work: As has been argued in the field of cross-cultural development (Bohn et al., 2024), while group-level analyses are important for highlighting how culture and development relate to one another, analyses within groups and at the level of the individual are needed to identify the sociocultural and ecological factors that shape developmental outcomes. In this respect, future research should expand not only beyond Western-biased samples but also beyond stark group-based comparisons, which can only offer limited conclusions about forces shaping development and the extent of its global diversity.

In summary, our findings contribute valuable cross-cultural insights into the socialization and development of prosocial behavior in early childhood. Specifically, assertive scaffolding was linked with higher infant helping across cultural contexts, suggesting that maternal insistence and clear requests for infant helping may play a crucial role in fostering early prosocial behavior. At the same time, our findings illustrate how maternal strategies are shaped by cultural and ecological variation with more assertive scaffolding in relational, low-resource contexts and deliberate scaffolding more prominent in the Untied Kingdom. More broadly, our results support the hypothesis that early prosocial development is shaped by socialization goals and practices grounded in caregiving environments, highlighting the importance of studying diverse sociocultural contexts to capture the vast diversity of human childrearing and learning environments and their influence on developmental outcomes.

## Supplementary Material

10.1037/dev0002159.supp

## Figures and Tables

**Table 1 tbl1:** Participant Demographics (Infant Age in Weeks and Sex by Time Point and Site)

Site	*N*	16 months		*N*	22 months	
		*M*_age_ (*SD*) in weeks	Infant sex		*M*_age_ (*SD*) in weeks	Infant sex
Rural Uganda	49	72.45 (1.55)	24 female, 25 male	48	97.21 (1.33)	23 female, 25 male
Urban Uganda	162	71.05 (2.44)	76 female, 86 male	135	96.63 (2.28)	61 female, 74 male
United Kingdom	62	71.76 (1.35)	29 female, 33 male	38	98.70 (2.27)	17 female, 21 male

**Table 2 tbl2:** Model Structures for Scaffolding and Helping Models

Type of scaffolding	Outcome	Fixed effect	Random effect
Assertive scaffolding	Requested helping	Assertive Scaffolding^a^ × Site^b^	Participant ID
Deliberate scaffolding^c^	Requested helping	Deliberate scaffolding^a^	Participant ID
Assertive scaffolding	Spontaneous helping	Assertive Scaffolding × Site; Assertive Scaffolding × Infant Age^d^	Participant ID
Deliberate scaffolding^c^	Spontaneous helping	Deliberate Scaffolding × Infant Age^d^	Participant ID
^a^ Coded on a 0–3 scale. ^b^ Site had three levels: rural Uganda, urban Uganda, the United Kingdom. ^c^ Deliberate scaffolding models were run for the U.K. sample only. ^d^ Infant age had two levels: 16 months and 22 months.			

**Table 3 tbl3:** Maternal Socialization Goals (Difference Scores), Percentage of Mothers Expecting Help at 16 and 22 Months, and Scaffolding Scores

Measure	Rural Uganda	Urban Uganda	United Kingdom
Socialization goals, *M* (*SD*)	−0.17 (0.62)	−0.74 (0.80)	0.47 (0.63)
% expecting help at 16 months	89.29%	98.28%	54.17%
% expecting help at 22 months	96.43%	98.10%	55.81%
Assertive scaffolding, *M* (*SD*)	2.06 (0.88)	1.77 (0.76)	1.23 (0.56)
Deliberate scaffolding, *M* (*SD*)	0.37 (0.60)	0.19 (0.50)	2.61 (0.64)
*Note*. Socialization goals score is between −4 and 4 (mean autonomous score minus mean relational score), with negative values indicating more relational socialization goals and positive values indicating more autonomous socialization goals. Scaffolding coded on a 0–3 scale.			

**Figure 1 fig1:**
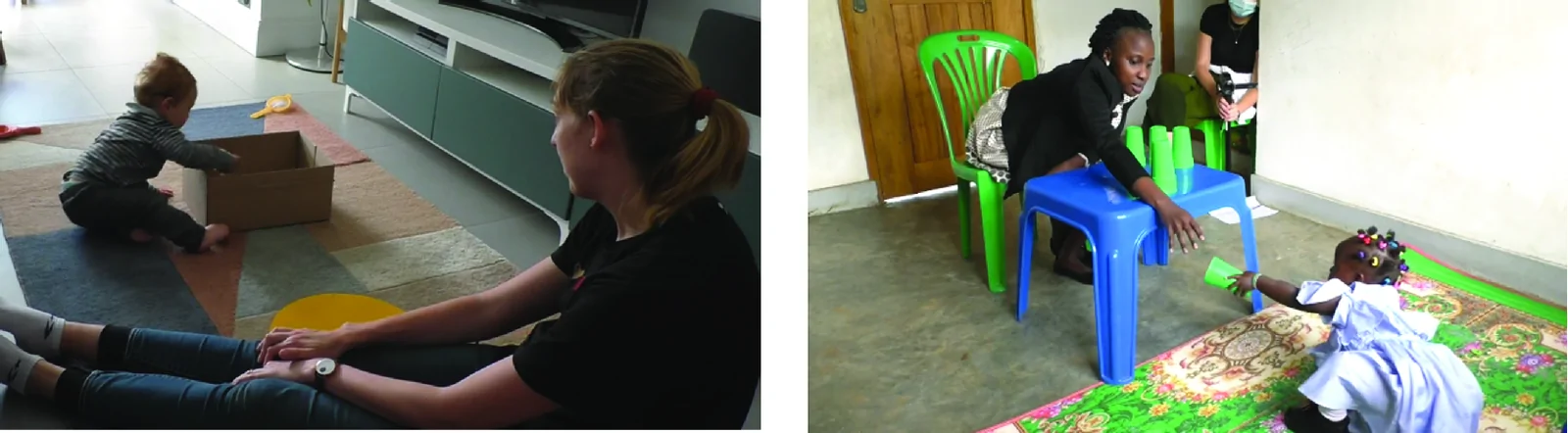
Experimental Setup for the Requested Helping Task (Left, U.K. Participant With Mother) and the Spontaneous Helping Task (Right, Urban Ugandan Participant With Experimenter) *Note*. Images published with permission. See the online article for the color version of this figure.

**Figure 2 fig2:**
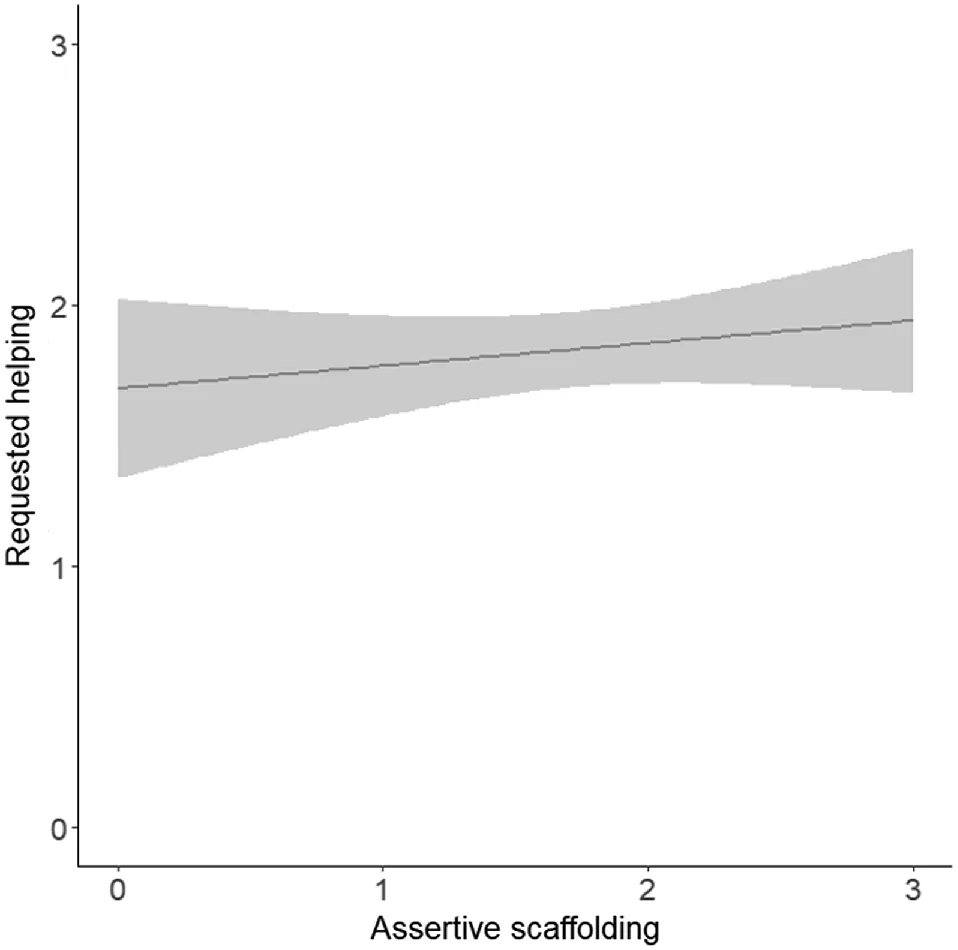
Relation Between Assertive Scaffolding (0–3) and Requested Helping (0–3) *Note*. *p* < .001. Regression line and 95% confidence interval.

**Figure 3 fig3:**
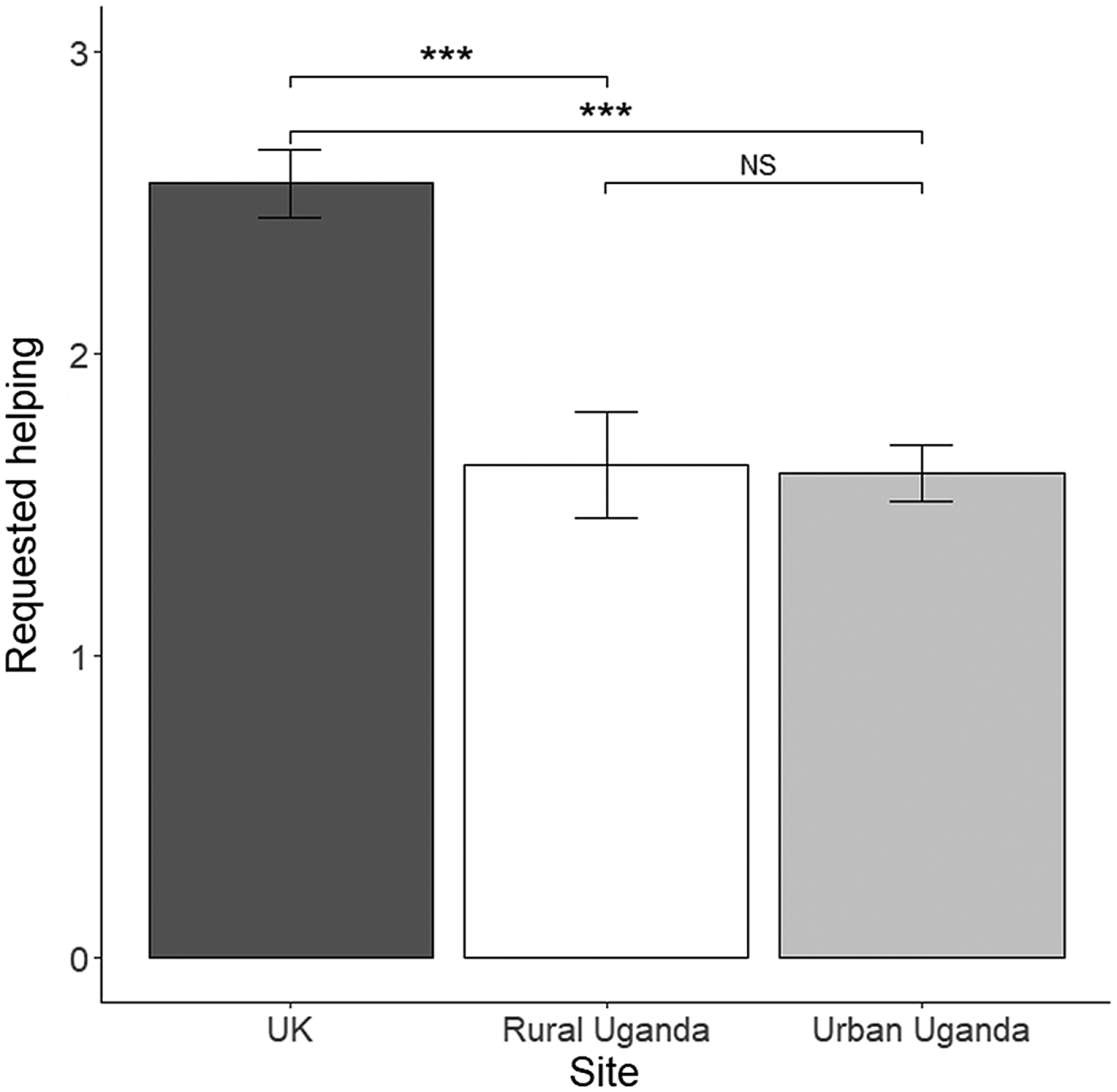
Mean Requested Helping (0–3) by Site (the United Kingdom, Rural Uganda, Urban Uganda) *Note*. Error bars represent the standard error of the mean. NS = not significant. *** *p* < .001.

**Figure 4 fig4:**
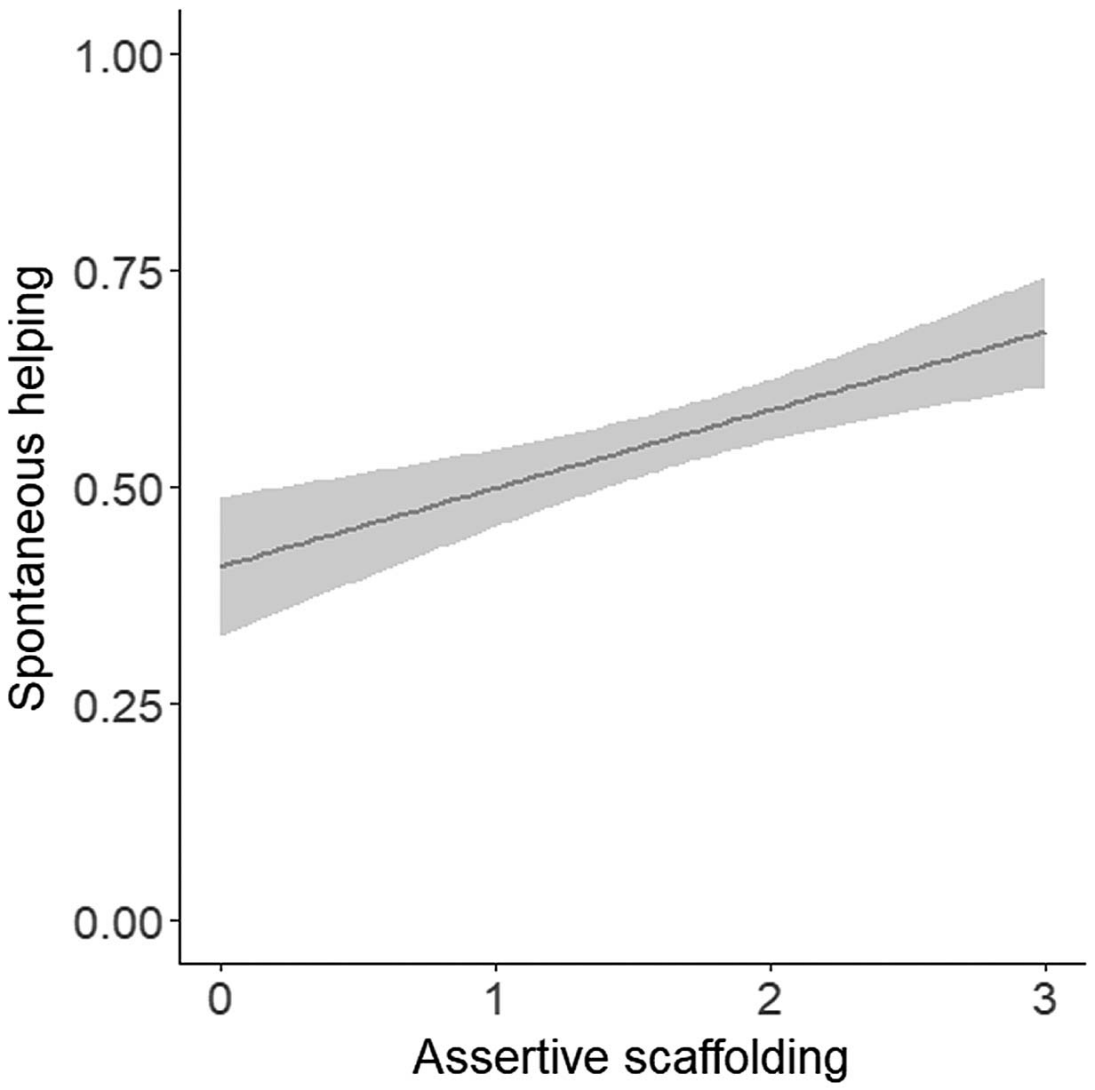
Relation Between Assertive Scaffolding (0–3) and Spontaneous Helping (0–1) *Note*. *p* = .030. Spontaneous helping is visualized as a proportion out of 1 (number of successful helping trials divided by number of valid trials) because not all infants completed five valid helping trials. Regression line and 95% confidence interval.

**Figure 5 fig5:**
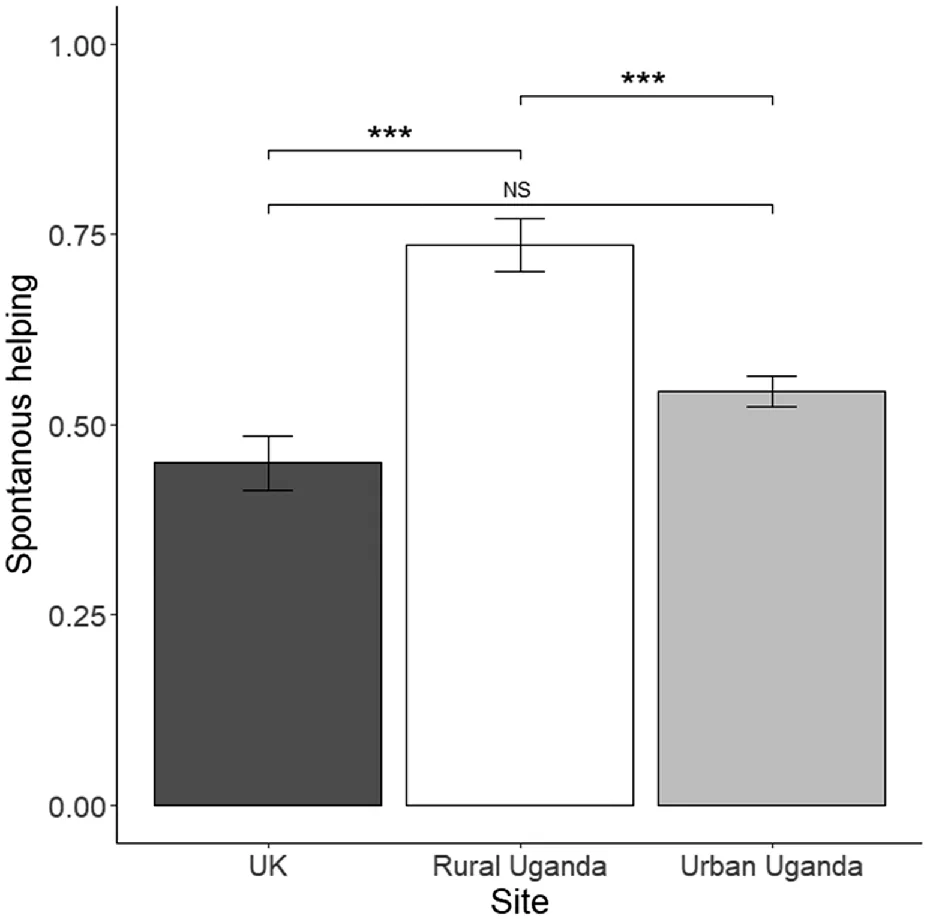
Mean Spontaneous Helping (0–1) by Site (the United Kingdom, Rural Uganda, Urban Uganda) *Note*. Spontaneous helping is visualized as a proportion out of 1 (number of successful helping trials divided by number of valid trials) because not all infants completed five valid helping trials. Error bars represent the standard error of the mean. NS = not significant. *** *p* < .001.

**Figure 6 fig6:**
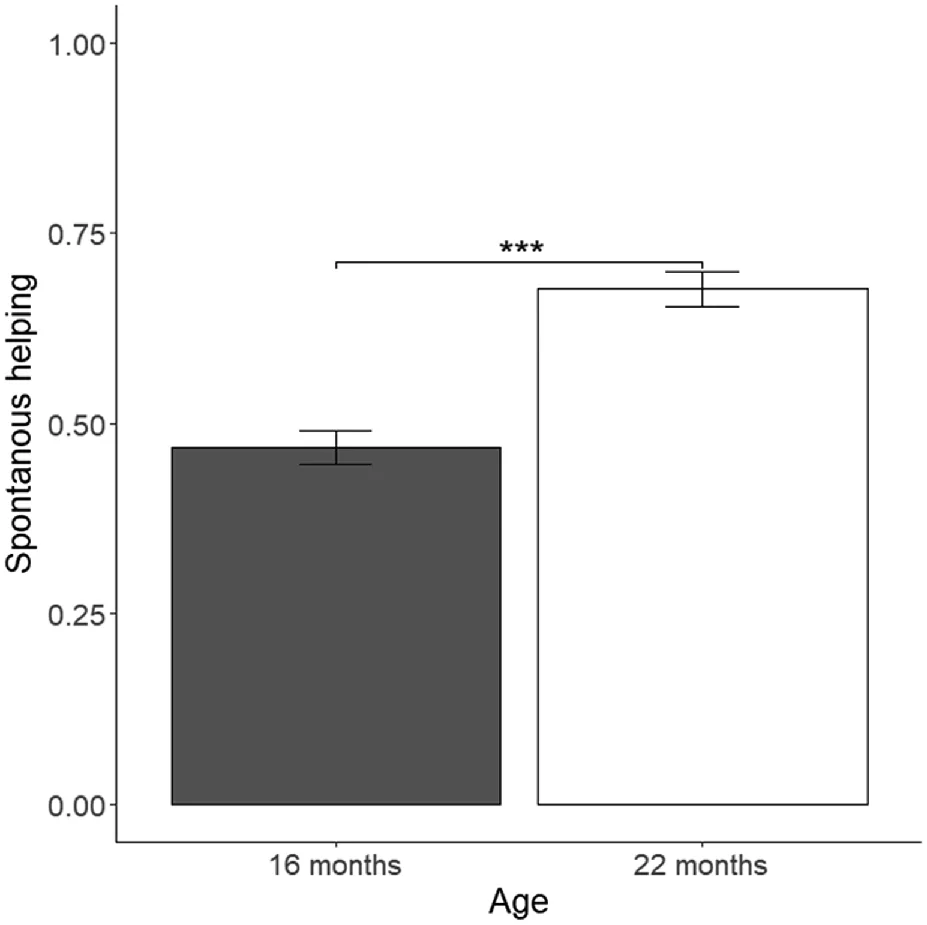
Spontaneous Helping (0–1) by Time Point (16 Months, 22 Months) *Note*. Spontaneous helping is visualized as a proportion out of 1 (number of successful helping trials divided by number of valid trials) because not all infants completed five valid helping trials. Error bars represent the standard error of the mean. *** *p* < .001.
