# Supplementary material for: Helping Infants to Help: Cultural Variation in Maternal Scaffolding and the Development of Prosocial Behavior
Source: Dev Psychol. 2026 May 7;62(12):2579–90. doi: 10.1037/dev0002159 (PMC13738043; doi:10.1037/dev0002159)
Supplement: Supplementary file 1 [file dev0002159_Supplemental_Materials.docx]

**Supplementary Materials: Full Model Estimates**

Where a reduced model (no interaction terms) was used to assess main effects of fixed effect variables (i.e., an interaction was not significant), the model estimates of this reduced model, not the full model, are presented. Binary variables were dummy coded, with the reference category indicated in parentheses.

**Table S1 Maternal Expectations for Infant Helping (Reduced Model)**

| Term | Estimate | SE | Lower CI | Upper CI | *χ^2^* | df | p |
| --- | --- | --- | --- | --- | --- | --- | --- |
| (Intercept) | -5.97 | 1.14 | -11.68 | -7.00 |  |  | ^(1)^ |
| Infant age (22 months) | -0.56 | 0.54 | -6.97 | 0.66 | 1.10 | 1 | .294 |
| Site (urban Uganda) | -1.38 | 1.05 | -1.45 | 0.68 | 19.26 | 2 | .191 |
| Site (UK) | 5.30 | 1.19 | 1.43 | 18.06 | 19.26 | 2 | <.001 |

^(1)^ H_0_ for the intercept is *intercept = 0.* The intercept represents the hypothetical value of the response assuming all predictors and random effects are zero, but the absolute baseline of the response is of no interest. It is not reported due to this limited interpretation.

**Table S2 Assertive Scaffolding and Requested Helping (Reduced Model)**

| Term | Estimate | SE | Lower CI | Upper CI | *χ^2^* | df | p |
| --- | --- | --- | --- | --- | --- | --- | --- |
| (Intercept) | 2.41 | 0.56 | 1.37 | 3.54 |  |  | ^(1)^ |
| Assertive scaffolding | 0.82 | 0.26 | 0.32 | 1.46 | 10.31 | 1 | .001 |
| Site (rural Uganda) | -3.77 | 0.72 | -5.41 | -2.40 | 50.42 | 2 | <.001 |
| Site (urban Uganda) | -3.55 | 0.58 | -4.87 | -2.43 | 50.42 | 2 | <.001 |

^(1)^ Not reported due to this limited interpretation.

**Table S3 Deliberate Scaffolding and Requested Helping (Full Model)**

| Term | Estimate | SE | Lower CI | Upper CI | *χ^2^* | df | p |
| --- | --- | --- | --- | --- | --- | --- | --- |
| (Intercept) | 7.91 | 3.55 | 5.63 | 14.43 |  |  | ^(1)^ |
| Deliberate scaffolding | -0.16 | 1.24 | -1.48 | 1.32 | 0.02 | 1 | .894 |

^(1)^ Not reported due to this limited interpretation.

**Table S4 Assertive Scaffolding and Spontaneous Helping (Reduced Model)**

| Term | Estimate | SE | Lower CI | Upper CI | *χ^2^* | df | p |
| --- | --- | --- | --- | --- | --- | --- | --- |
| (Intercept) | -1.27 | 0.27 | -1.80 | -0.74 |  |  | ^(1)^ |
| Infant age (22 months) | 1.17 | 0.10 | 0.97 | 1.38 | 131.62 | 1 | <.001 |
| Assertive scaffolding | 0.39 | 0.13 | 0.13 | 0.64 | 8.52 | 1 | .004 |
| Site (rural Uganda) | 1.32 | 0.34 | 0.66 | 1.98 | 17.30 | 2 | <.001 |
| Site (urban Uganda) | 0.34 | 0.26 | -0.17 | 0.84 | 17.30 | 2 | <.001 |

^(1)^ Not reported due to this limited interpretation.

**Table S5 Deliberate Scaffolding and Spontaneous Helping (Full Model)**

| Term | Estimate | SE | Lower CI | Upper CI | *χ^2^* | df | p |
| --- | --- | --- | --- | --- | --- | --- | --- |
| (Intercept) | -1.00 | 0.95 | -2.87 | 0.87 |  |  | ^(1)^ |
| Infant age (22 months) | 0.93 | 0.23 | 0.47 | 1.38 | 15.88 | 1 | <.001 |
| Deliberate scaffolding | 0.11 | 0.35 | -0.58 | 0.79 | 0.10 | 1 | .758 |

^(1)^ Not reported due to this limited interpretation.
